# Theoretical Thermal-Mechanical Modelling and Experimental Validation of a Three-Dimensional (3D) Electrothermal Microgripper with Three Fingers

**DOI:** 10.3390/mi12121512

**Published:** 2021-12-04

**Authors:** Guoning Si, Liangying Sun, Zhuo Zhang, Xuping Zhang

**Affiliations:** 1 School of Health Science and Engineering, University of Shanghai for Science and Technology, Shanghai 200093, China; gnsi@usst.edu.cn (G.S.); sly19960721@163.com (L.S.); 2 School of Modern Posts, Xi’an University of Posts and Telecommunications, Xi’an 710061, China; zhangzhuozz123@126.com; 3 Department of Mechanical and Production Engineering, Aarhus University, 8200 Aarhus, Denmark

**Keywords:** thermal-mechanical modeling, 3D U-shaped electrothermal actuator, bi-directional Z-shaped electrothermal actuator, 3D microgripper, parameter analysis, micro-manipulation

## Abstract

This paper presents the theoretical thermal-mechanical modeling and parameter analyses of a novel three-dimensional (3D) electrothermal microgripper with three fingers. Each finger of the microgripper is composed of a bi-directional Z-shaped electrothermal actuator and a 3D U-shaped electrothermal actuator. The bi-directional Z-shaped electrothermal actuator provides the rectilinear motion in two directions. The novel 3D U-shaped electrothermal actuator offers motion with two degrees of freedom (DOFs) in the plane perpendicular to the movement of the Z-shaped actuator. As a result, each finger possesses 3D mobilities with three DOFs. Each beam of the actuators is heated externally with polyimide films. In this work, the static theoretical thermal-mechanical model of the 3D U-shaped electrothermal actuator is established. Finite-element analyses and experimental tests are conducted to verify and validate the model. With this model, parameter analyses are carried out to provide insight and guidance on further improving the 3D U-shaped actuator. Furthermore, a group of micro-manipulation experiments are conducted to demonstrate the flexibility and versality of the 3D microgripper on manipulate different types of small/micro-objects.

## 1. Introduction

Micro-manipulation techniques have a variety of potential applications such as micro-assembly [1,2,3], micro-injection [4,5,6], Pharmaceutical Development [7,8], artificial insemination [9,10] and micro-surgery [11,12,13], etc. To achieve micro-manipulation of different micro-objects, various types of micro-tools have been developed. The micropipette, which is widely used for manipulation of biological cells, relies on the negative pressure to suck the cell and the positive air pressure to release and even rotate a cell [14,15,16,17]. However, the air pressure is considerably difficult to control, which often results in damages of the cells. In addition, the micropipette is inapplicable to situations where the micro-object is immersed in the liquid. Another important micro-tool category involves the microgripper, which uses mechanical force and displacement produced by a pair of micro-fingers to manipulate micro-objects. The direct-contact manner for micro-manipulations has demonstrated its wide adaptability for a variety of micro-objects such as wires, animal hairs, biological cells, and iron spheres [18,19,20,21,22,23]. In the authors’ previous work, the geometrical confine to the zebrafish embryo created by the jaws results in less deformation of the cell during micro-injection [24]. It is also found that among other types of microgrippers, the electrothermal actuated microgrippers are compact, easy to control, and can provide moderate response speed without an overshoot [25,26,27,28]. These characteristics render it suitable for almost all micro-manipulation applications. In addition, the electrothermal microgripper work with internal or external heating of the beams, which imparts the microgripper large room for more complicated designs [29,30,31]. However, both the micropipette and the microgripper only offer limited DOFs. The micropipette works simply with suck and blow of air, and the microgripper that is usually two-fingered type, can only offer movement of opening and closing the jaws. Moreover, the microgripper designs are confined to in-plane structure with a pair of fingers, and are unsuited to 3D micro-manipulations like 3D orientating a zebrafish embryo for microinjection. Though researchers have proposed multi-fingered microgripper, these microgrippers only involve single DOF on each finger, and are unsuited for complicated operations like rotations. 

In the authors’ preliminary work [32], a 3D microgripper is proposed, which consists of three fingers. Each finger has three DOFs provided by a V-shaped electrothermal actuator and a 3D electrothermal U-shaped actuator. The 3D microgripper is proved to function in picking, holding, rotating a micro-object in 3D space, and releasing. Compared with the previous work, this paper adds the static theoretical modeling of the 3D U-shaped actuator and verifies the correctness of the theoretical model through finite element (FE) simulation and test. Furthermore, the parameter analysis of the 3D U-shaped actuator is added. Finally, the structure of the microgripper changes, which the V-shaped actuator is replaced by the bi-directional Z-shaped actuator. 

The advantage of this article is that the statics theory model of the 3D U-shaped actuator can be used to calculate the output displacement at different temperatures, and its accuracy can be verified by FE simulation and testing. In addition, many research efforts have been made towards the statics theoretical modeling of the electrothermal micro-actuators and microgrippers [33,34]. Nevertheless, there is no report on the statics theoretical modeling of 3D electrothermal actuators in the literature. For the 3D U-shaped actuator, the connection relationship of the beams occurs in 3D space and makes the statics theoretical modelling much more challenging. The parameter analysis of the 3D U-shaped actuator is carried out to guide the dimension optimization of microgripper, so that it can achieve better stability and greater displacement in the future work. In this paper, the bi-directional Z-shaped actuator has bi-directional active motion. The bi-directional Z-shaped actuator can increase the flexibility and more convenient control of the microgripper, and it can realize more accurate operation in micro operation. However, the V-shaped actuator in previous work can only accomplish bi-direction passive motion when heating, which in a great extent has limited the flexibility of manipulation and increased controlling challenges [35,36,37,38].

This work is a follow-up research effort based on the 3D microgripper we proposed previously. The major difference is that the V-shaped actuator is replaced by a bi-directional Z-shaped actuator to impart a bi-directional actuation ability. The bi-directional Z-shaped actuator was proposed in published papers and has potential applications where bi-directional actuation is required such as micro-stages [39]. Another important difference about the 3D U-shaped actuator is that the four pairs of beams employed in this work, instead of four single beams, are arranged on the four sides of a rectangular base. This helps increase the stability of actuation. For the design and control of the 3D microgripper, the static thermal-mechanical model of the 3D U-shaped actuator is established, and then is verified via finite element simulations and further validated with experimental testing. Parameter analyses are also carried out to provide insight and guidance on further improving the design. Finally, micro-manipulations of a micro-ball and a zebrafish embryo are conducted, which involves picking, holding, 3D orientating and releasing, etc. Table 1 summarizes the challenges in this paper and how to address them.

This work is organized as follows. In Section 2, the structural designs of the 3D U-shaped actuator and the bi-directional Z-shaped actuator are presented. In Section 3, the static theoretical model of the 3D U-shaped electrothermal actuator is established followed by finite-element simulations in Section 4. Then, in Section 5, experimental testing is carried out to further validate the model. Based on the theoretical model, detailed parameter analyses are carried out in Section 6. In Section 7, dynamic and static performance of both the Z-shaped and 3D U-shaped actuators are tested. Finally, micro-manipulations of a micro ball and a zebrafish embryo are conducted including picking, holding, lifting, 3D orientating, and releasing operations. 

## 2. Structural Design of the 3D U-Shaped Electrothermal Actuator

**Figure 1:**
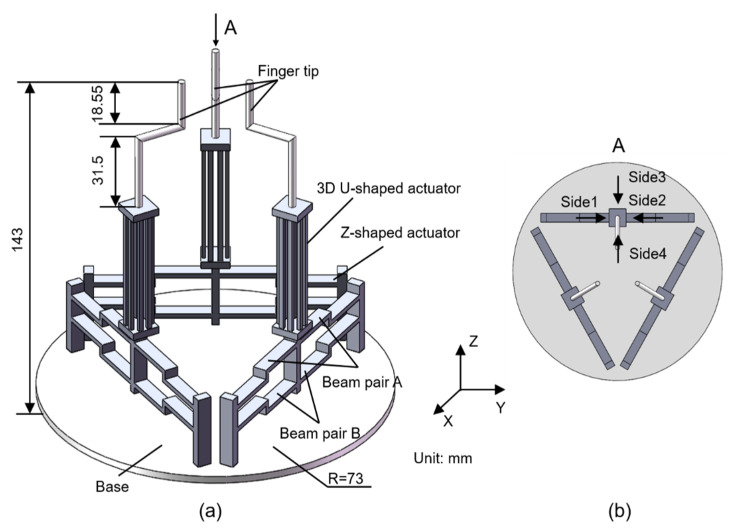
Structure of the 3D electrothermal microgripper: (**a**) general view, (**b**) top view.

The schematic diagram of the three-dimensional(3D) electrothermal microgripper proposed in this paper is shown in Figure 1a. The microgripper has three identical fingers distributed evenly on the base, as shown in Figure 1b. The dexterous 3D manipulation capabilities are realized with the combination of a bi-directional Z-shaped electrothermal actuator and a 3D U-shaped electrothermal actuator proposed in this work. It is named as the 3D U-shaped actuator in order to be differentiated from the common U-shaped actuator, which is essentially the 2D structure possessing only planar actuation capability. In contrast to the traditional electrothermal micro actuators that are fabricated via micromachining process, the 3D microgripper is fabricated using metal 3D printing resulting in larger size. Because metals have extremely great conductivity, applying a voltage will result in short circuit. Therefore, external heat source is employed using the polyimide films adhered on the beam surface. Detailed discussions of the polyimide film are presented in Section 5. AlSi10Mg is selected as the microgripper material for metal 3D printing with consideration of its large thermal conductivity (203 W/(m·K)), large thermal expansion coefficient (23 × 10^−6^/°C), and small Young’s modulus (6 × 10^10^ Pa).

**Figure 2:**
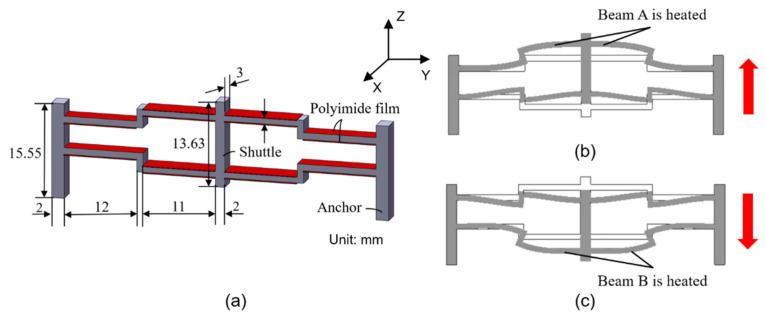
Structure of Z-shaped electrothermal actuator. (**a**) structure and dimensions of the bi-directional Z-shaped actuator; (**b**) When the beam A of the Z-shaped actuator are heated; (**c**) When the beam B of the Z-shaped actuator are heated.

The bi-directional design of the Z-shaped actuator that imparts bi-directional actuation capabilities to the actuator stems from the fact that the stiffness of the Z-shaped beam is much smaller compared with the V-shaped counterpart [40]. The principle of the bi-directional Z-shaped actuators is straightforward: when the upper Z-shaped beam (i.e., the Z-shaped beam farthest to the base) is heated, whereas the lower Z-shaped beam is unheated, the middle shuttle will be pushed forward due to symmetric thermal expansion of the upper Z-shaped beam and vice versa, as shown in Figure 2b,c. In order to refer to each of the beams conveniently, the upper and lower Z-shaped beams are denoted as beam A and beam B, respectively. Clearly, heating the beam A or the beam B will result in the Z-shaped actuator to move in the opposite direction, which leads to one DOF.

**Figure 3:**
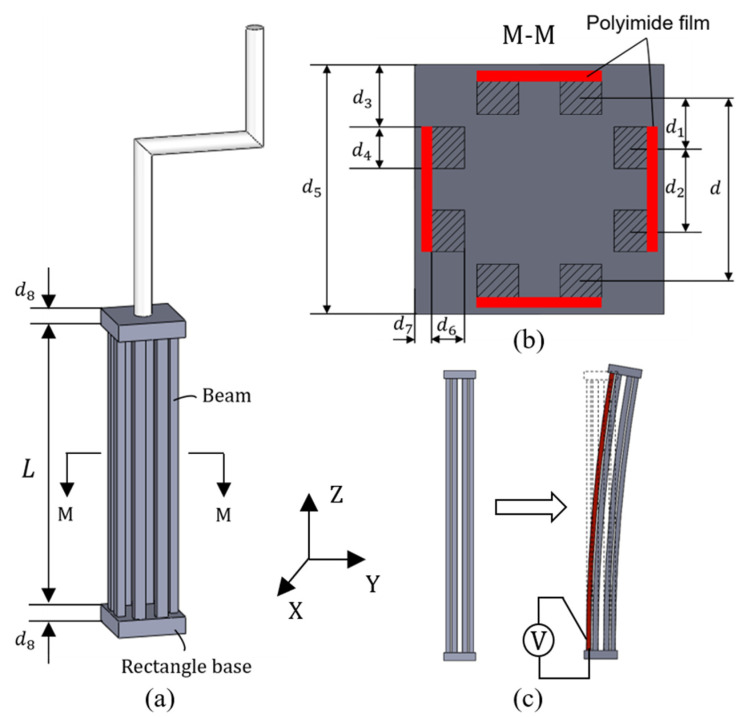
Detailed structure design of 3D U-shaped electrothermal actuator. (**a**) structure of 3D U-shaped electrothermal actuator; (**b**) the sectional view of 3D U-shaped electrothermal actuator; (**c**) deformation of the 3D U-shaped electrothermal actuator.

The detailed structure of the 3D U-shaped electrothermal actuators is depicted in Figure 3a,b (the cross-sectional view). Each 3D U-shaped actuator consists of four pairs of beams located each sides of the rectangular base. When one of the four pairs of beams are heated, the elevated temperature causes elongation of the heated pair of beams. As a result, the other three pairs of beams are pushed to deform thus generating displacement, as shown in Figure 3c. Since the 3D U-shaped beam are made up of four pairs of beams, each of which is located at one side of the rectangular base, the 3D U-shaped actuator has two DOFs. 

The dimensions of the primarily designed 3D U-shaped actuator are defined and listed in Table 2. Dimensions of the Z-shaped actuator are indicated in Figure 2a. The dimensions are chosen considering the fabrication restrictions and based on the finite-element analyses (presented in Section 4).

## 3. Theoretical Modeling of the 3D U-Shaped Actuator

The 3D U-shaped actuator consists of two parts, that is, the polyimide films and the actuator beams. The polyimide films, i.e., the electro-thermal part, convert input voltage to thermal energy leading to temperature rise of the films and then the actuator beams via thermal conduction. The actuator beams, i.e., the thermal-mechanical part, then convert the temperature rise to the elongation of the heated beams, thus generating displacement and force as the output. As a result, in order to generate larger displacement, the thermal-mechanical model should be established first. 

**Figure 4:**
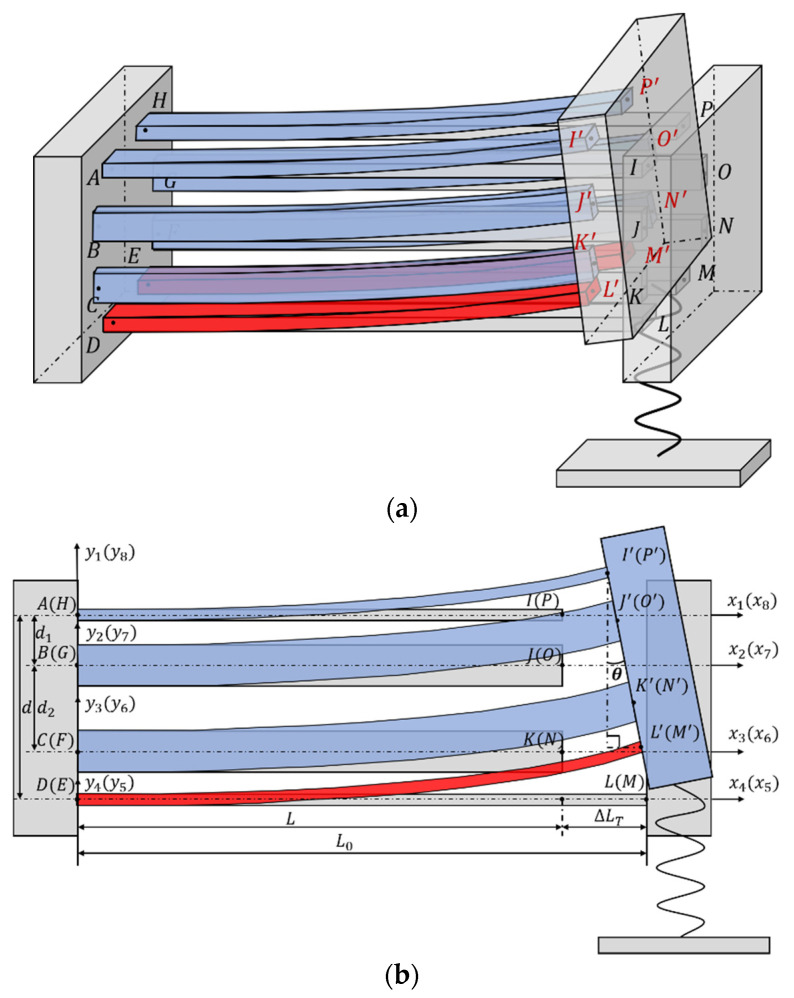
Structure analysis of the 3D U-shaped actuator. (**a**) the general view; (**b**) the front view.

The 3D U-shaped electrothermal actuator is shown in Figure 4a, where the red beams represent the heated beams. In order to derive the deformation of the structure, the other three pairs of beams are assumed to be unconnected to the right end block, so that the heating beam can be elongated freely for given temperature rises, as shown in Figure 3b (the grey beams). Then, the beam is connected to the right end block with the same connection relationships as the original. Due to deformation coordination relationships between the beams and the right block, the extra elongated beams’ length will cause the whole structure to bend. For applications like biological cell manipulations, the cells are highly deformable and light weight with limited stiffness; as a result, the force a cell exerted on the microgripper tips during manipulation is small and can thus be ignored. In this case, the microgripper is required to generate large displacement to allow for large cell deformations. However, for some applications like micro-assembly, the micro-objects are usually hard and much heavier with large stiffness. Hence, the microgripper needs to generate large force to overcome the gravity of the micro object while smaller output displacement of the gripping fingers is sufficient. In order to design a microgripper with both large output displacement and force, a spring is added to the end of the 3D U-shaped actuator tip to simulate the object stiffness. 

As shown in Figure 4b, the original beam length is L, the elongated beam length is $\Delta L_{T}$, and the final beam length of the heated beam is denoted as $L_{0}$. The theoretical thermal-mechanical model is established based on the following assumptions: (1) The angles between the beams and the base at the right end remains as a right angle; (2) The right end base is assumed to be rigid, only the beam is deformable; (3) Because of symmetry, the bending of the actuator only occurs in the plane as shown in Figure 4b.

**Figure 5:**
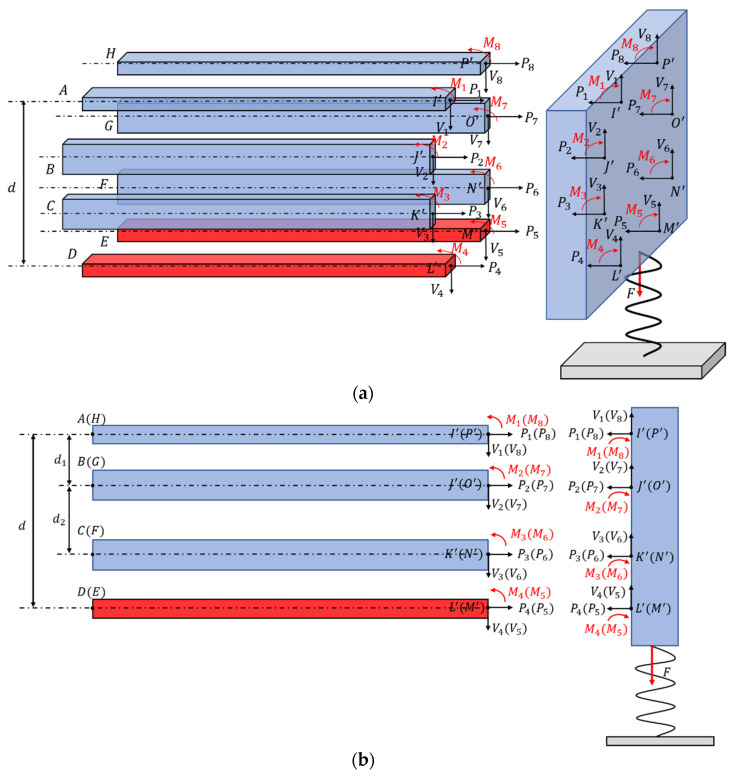
Free-body diagram used in determining the bending moment, axial force, and the shear force acting on the beams: (**a**) the general view; (**b**) the front view.

With the assumption of one-dimensional expansion of the beam, the elongated length $\Delta L_{T}$ of the beam is calculated as

(1) $$\Delta L_{T}=\int_{0}^{L}\alpha \left(T-T_{0}\right)dx$$

where α is coefficient of thermal expansion of the beams, $T_{0}$ is initial temperature of the beams, T is the real-time temperature of the beams, and L is original beams length. When the heated beams are elongated, the corresponding moment and force are generated at the right end of the beams, as illustrated in Figure 5, where $P_{i}$, $V_{i}$, and $M_{i}$ (i=1, 2, 3, 4, 5, 6, 7, 8) are axial force, shear force and bending moment, respectively. Because the right end block is assumed rigid, an equilibrium system of equations of force and moment can be derived as

(2) $$P_{1}+P_{2}+P_{3}+P_{4}+P_{5}+P_{6}+P_{7}+P_{8}=0$$

(3) $$V_{1}+V_{2}+V_{3}+V_{4}+V_{5}+V_{6}+V_{7}+V_{8}-F=0$$

(4) $$P_{2}\cdot d_{1}+P_{3}\cdot \left(d_{1}+d_{2}\right)+P_{4}\cdot d+P_{5}\cdot d+P_{6}\cdot \left(d_{1}+d_{2}\right)+P_{7}\cdot d_{1}+M_{1}+M_{2}+M_{3}+M_{4}+M_{5}+M_{6}+M_{7}+M_{8}=0$$

(5) $$P_{1}\cdot d_{1}+P_{3}\cdot d_{2}+P_{4}\cdot \left(d_{1}+d_{2}\right)+P_{5}\cdot \left(d_{1}+d_{2}\right)+P_{6}\cdot d_{2}+P_{8}\cdot d_{1}=0$$

(6) $$V_{1}\cdot d_{1}+V_{3}\cdot d_{2}+V_{4}\cdot \left(d_{1}+d_{2}\right)+V_{5}\cdot \left(d_{1}+d_{2}\right)+V_{6}\cdot d_{2}+V_{8}\cdot d_{1}-F=0$$

According to Equations (A1) and (A2) in the Appendix A, the elongation of eight beams is as follows: $\Delta u_{AI}=\frac{P_{1}L}{EA}$, $\Delta u_{BJ}=\frac{P_{2}L}{EA}$, $\Delta u_{CK}=\frac{P_{3}L}{EA}$, $\Delta u_{DL}=\frac{P_{4}L_{0}}{EA}$, $\Delta u_{HP}=\frac{P_{5}L}{EA}$, $\Delta u_{GO}=\frac{P_{6}L}{EA}$, $\Delta u_{FN}=\frac{P_{7}L}{EA}$ and $\Delta u_{EM}=\frac{P_{8}L_{0}}{EA}$, where $L_{0}=L+\Delta L_{T}$, E and A are the Young’s modulus and the cross-sectional area of the beam, respectively. The resultant length of the beams $AI^{\prime }$, $BJ^{\prime }$, $CK^{\prime }$,$DL^{\prime }$, $EM^{\prime }$, $FN^{\prime }$,$GO^{\prime }$ and $HP^{\prime }$ can be obtained as follows: $u_{AI^{\prime }}=L+\Delta u_{AI}$, $u_{BJ^{\prime }}=L+\Delta u_{BJ}$, $u_{CK^{\prime }}=L+\Delta u_{CK}$, $u_{DL^{\prime }}=L+\Delta u_{DL}$, $u_{HP^{\prime }}=L+\Delta u_{HP}$, $u_{GO^{\prime }}=L+\Delta u_{GO}$, $u_{FN^{\prime }}=L+\Delta u_{FN}$, and $u_{EM^{\prime }}=L+\Delta u_{EM}$. According to Equation (A8) and (A9) in the Appendix A, the deflection and slope of the deflection curve at the free end can be obtained, as follow: 

$w\left(u_{AI^{\prime }}\right)=\frac{M_{1}u_{AI^{\prime }}{}^{2}}{2EI_{AI^{\prime }}}-\frac{V_{1}u_{AI^{\prime }}{}^{3}}{3EI_{AI^{\prime }}}$, $w\left(u_{BJ^{\prime }}\right)=\frac{M_{2}u_{BJ^{\prime }}{}^{2}}{2EI_{BJ^{\prime }}}-\frac{V_{2}u_{BJ^{\prime }}{}^{3}}{3EI_{BJ^{\prime }}}$, $w\left(u_{CK^{\prime }}\right)=\frac{M_{3}u_{CK^{\prime }}{}^{2}}{2EI_{CK^{\prime }}}-\frac{V_{3}u_{CK^{\prime }}{}^{3}}{3EI_{CK^{\prime }}}$, $w\left(u_{DL^{\prime }}\right)=\frac{M_{4}u_{DL^{\prime }}{}^{2}}{2EI_{DL^{\prime }}}-\frac{V_{4}u_{DL^{\prime }}{}^{3}}{3EI_{DL^{\prime }}}$, $w\left(u_{EM^{\prime }}\right)=\frac{M_{5}u_{EM^{\prime }}{}^{2}}{2EI_{EM^{\prime }}}-\frac{V_{5}u_{EM^{\prime }}{}^{3}}{3EI_{EM^{\prime }}}$, $w\left(u_{FN^{\prime }}\right)=\frac{M_{6}u_{FN^{\prime }}{}^{2}}{2EI_{FN^{\prime }}}-\frac{V_{6}u_{FN^{\prime }}{}^{3}}{3EI_{FN^{\prime }}}$, $w\left(u_{GO^{\prime }}\right)=\frac{M_{7}u_{GO^{\prime }}{}^{2}}{2EI_{GO^{\prime }}}-\frac{V_{7}u_{GO^{\prime }}{}^{3}}{3EI_{GO^{\prime }}}$, $w\left(u_{HP^{\prime }}\right)=\frac{M_{8}u_{HP^{\prime }}{}^{2}}{2EI_{HP^{\prime }}}-\frac{V_{8}l_{HP^{\prime }}{}^{3}}{3EI_{HP^{\prime }}}$, $w^{\prime }\left(u_{AI^{\prime }}\right)=\frac{M_{1}u_{AI^{\prime }}}{EI_{AI^{\prime }}}-\frac{V_{1}u_{AI^{\prime }}{}^{2}}{2EI_{AI^{\prime }}}$, $w^{\prime }\left(u_{BJ^{\prime }}\right)=\frac{M_{2}u_{BJ^{\prime }}}{EI_{BJ^{\prime }}}-\frac{V_{2}u_{BJ^{\prime }}{}^{2}}{2EI_{BJ^{\prime }}}$, $w^{\prime }\left(u_{CK^{\prime }}\right)=\frac{M_{3}u_{CK^{\prime }}}{EI_{CK^{\prime }}}-\frac{V_{3}u_{CK^{\prime }}{}^{2}}{2EI_{CK^{\prime }}}$, $w^{\prime }\left(u_{DL^{\prime }}\right)=\frac{M_{4}u_{DL^{\prime }}}{EI_{DL^{\prime }}}-\frac{V_{4}u_{DL^{\prime }}{}^{2}}{2EI_{DL^{\prime }}}$, $w^{\prime }\left(u_{EM^{\prime }}\right)=\frac{M_{5}u_{EM^{\prime }}}{EI_{EM^{\prime }}}-\frac{V_{5}u_{EM^{\prime }}{}^{2}}{2EI_{EM^{\prime }}}$, $w^{\prime }\left(u_{FN^{\prime }}\right)=\frac{M_{6}u_{FN^{\prime }}}{EI_{FN^{\prime }}}-\frac{V_{6}u_{FN^{\prime }}{}^{2}}{2EI_{FN^{\prime }}}$, $w^{\prime }\left(u_{GO^{\prime }}\right)=\frac{M_{7}u_{GO^{\prime }}}{EI_{GO^{\prime }}}-\frac{V_{7}u_{GO^{\prime }}{}^{2}}{2EI_{GO^{\prime }}}$ and $w^{\prime }\left(u_{HP^{\prime }}\right)=\frac{M_{8}u_{HP^{\prime }}}{EI_{HP^{\prime }}}-\frac{V_{8}u_{HP^{\prime }}{}^{2}}{2EI_{HP^{\prime }}}$.

Since the right end block is assumed rigid, the slopes of each beams at the free end are the same, which gives

(7) $$w^{\prime }\left(u_{AH^{\prime }}\right)=w^{\prime }\left(u_{BG^{\prime }}\right)=tan\theta \approx \theta$$

(8) $$w^{\prime }\left(u_{BG^{\prime }}\right)=w^{\prime }\left(u_{CF^{\prime }}\right)$$

(9) $$w^{\prime }\left(u_{CF^{\prime }}\right)=w^{\prime }\left(u_{DE^{\prime }}\right)$$

(10) $$w^{\prime }\left(u_{DE^{\prime }}\right)=w^{\prime }\left(u_{EM^{\prime }}\right)$$

(11) $$w^{\prime }\left(u_{EM^{\prime }}\right)=w^{\prime }\left(u_{FN^{\prime }}\right)$$

(12) $$w^{\prime }\left(u_{FN^{\prime }}\right)=w^{\prime }\left(u_{GO^{\prime }}\right)$$

(13) $$w^{\prime }\left(u_{GO^{\prime }}\right)=w^{\prime }\left(u_{HP^{\prime }}\right)$$

The deformation and connection relationship for the beams are shown in Figure 4b, which can be described with the equations as

(14) $$d\cdot cos\theta -w\left(u_{AI^{\prime }}\right)=d-w\left(u_{DL^{\prime }}\right)$$

(15) $$d\cdot cos\theta -w\left(u_{AI^{\prime }}\right)=d-w\left(u_{EM^{\prime }}\right)$$

(16) $$\left(d_{1}+d_{2}\right)\cdot cos\theta -w\left(u_{AI^{\prime }}\right)=d_{1}+d_{2}-w\left(u_{CK^{\prime }}\right)$$

(17) $$\left(d_{1}+d_{2}\right)\cdot cos\theta -w\left(u_{AI^{\prime }}\right)=d_{1}+d_{2}-w\left(u_{FN^{\prime }}\right)$$

(18) $$d_{1}\cdot cos\theta -w\left(u_{AI^{\prime }}\right)=d_{1}-w\left(u_{BJ^{\prime }}\right)$$

(19) $$d_{1}\cdot cos\theta -w\left(u_{AI^{\prime }}\right)=d_{1}-w\left(u_{GO^{\prime }}\right)$$

(20) $$\Delta u_{AI}+d\cdot sin\theta =\Delta L_{T}+\Delta u_{DL}$$

(21) $$\Delta u_{AI}+d\cdot sin\theta =\Delta L_{T}+\Delta u_{EM}$$

(22) $$\Delta u_{AI}+\left(d_{1}+d_{2}\right)\cdot sin\theta =\Delta u_{CK}$$

(23) $$\Delta u_{AI}+\left(d_{1}+d_{2}\right)\cdot sin\theta =\Delta u_{FN}$$

(24) $$\Delta u_{AI}+d_{1}\cdot sin\theta =\Delta u_{BJ}$$

(25) $$\Delta u_{AI}+d_{1}\cdot sin\theta =\Delta u_{GO}$$

**Figure 6:**
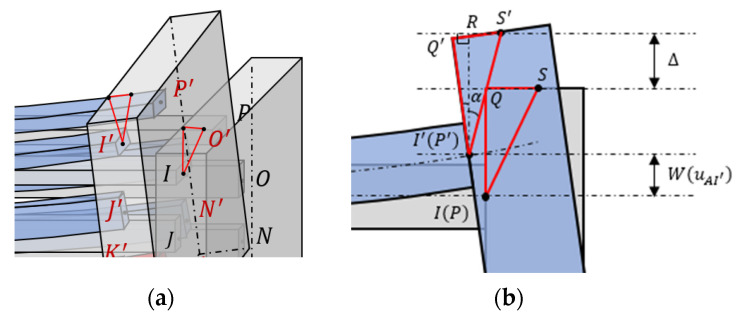
The geometric relationship between $w\left(u_{AI^{\prime }}\right)$ and output displacement of the 3D U-shaped actuator Δ. (**a**) Stereoscopic view; (**b**) the flat view.

Solving the system of Equations (2)–(25) using the Newton iteration method, all the unknown variables as well as the deflection of the beam $w\left(u_{AI^{\prime }}\right)$ at I can be obtained. In order to facilitate measurement of the output displacement in simulations and experiments, the output displacement of the 3D U-shaped actuator Δ is defined as the deflection of the beam at point S instead of I as shown in Figure 6, where S is the center of the upper surface of the base.

Obviously,

(26) $$\Delta =\bar{S^{\prime }I^{\prime }}\cdot cos\alpha -\bar{QI}+w\left(u_{AI^{\prime }}\right)$$

where

(27) $$\bar{S^{\prime }I^{\prime }}=\bar{SI}=\sqrt{{\bar{QI}}^{2}+{\bar{QS}}^{2}}$$

(28) $$\alpha =\cos^{-1}\frac{\bar{IQ}}{\bar{SI}}-\theta$$

## 4. Finite Element Analysis of the 3D U-Shaped Actuator

**Figure 7:**
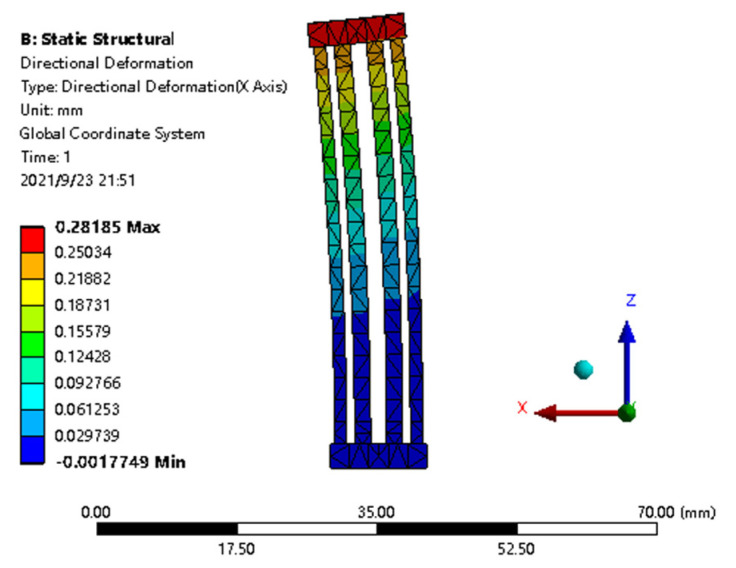
Finite element analysis of the 3D U-shaped electrothermal actuator.

In this section, finite element analysis (using ANSYS Workbench) is used to verify the established model, as shown in Figure 7. The material properties and actuator dimensions are given in Table 2 and Section 2, respectively. Firstly, the geometric model of the actuator is established and the material parameters of the actuator are defined. Besides, the actuator is meshed by intelligent meshing and the mesh type is tetrahedron. Furthermore, the initial temperature is the same as room temperature, which is 300 K and the constraint condition of fixing rectangular base on one side of the 3D U-shaped actuator. Then, a temperature load is applied to the beams of the 3D U-shaped actuator. Finally, the temperature distribution and direction deformation of the 3D U-shaped actuator are solved. In the simulations, the elastic modulus E of the end block of the 3D U-shaped actuator is set big enough to be in line with the assumption of rigid end block. The simulation begins by applying a temperature input to the beams to be heated. Three cases are simulated here. (1) No external load is applied on the end block; (2) a constant force is applied to the end block; (3) a spring is attached on the end block. It is seen from Table 3 that the analytical prediction for the output displacement agrees well with the simulation results for a wide range of temperature inputs. The deviation $\delta_{1}$% between the analytical and simulation results is calculated as

(29) $$\delta_{1}\%=\frac{d_{Analytical}-d_{Ansysy}}{d_{Analytical}}\times 100\%,$$

## 5. Experimental Validation of the Theoretical Model

**Figure 8:**
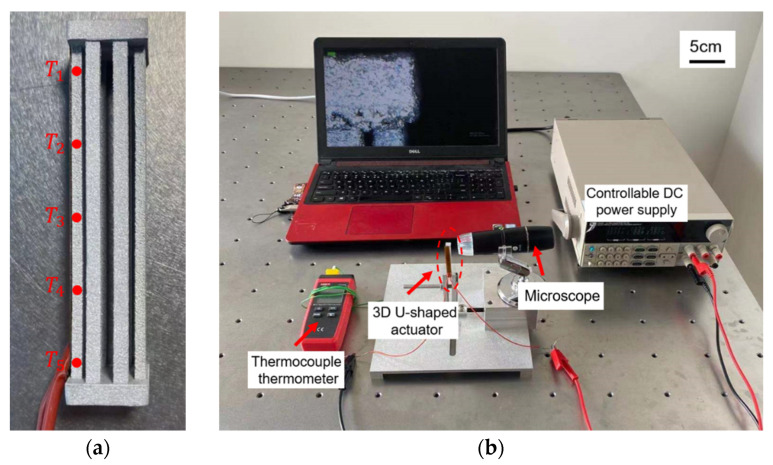
Experimental setup. (**a**) the fabricated 3D U-shaped electrothermal actuator with the polyimide film; (**b**) measuring devices.

**Figure 9:**
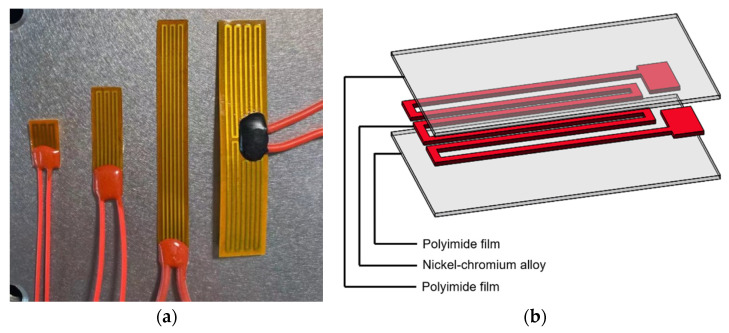
The polyimide films. (**a**) The polyimide electrothermal film; (**b**) Structure of polyimide electrothermal film.

In this section, the theoretical model of the 3D U-shaped electrothermal actuator is further validated via experimental testing. The experimental setup is shown in Figure 8. In both analytical model and simulations, average temperature rise is directly applied to the beams. In contrast, an indirect temperature elevating mechanism is employed with external heat source produced by the polyimide film, as shown in Figure 8a. The polyimide film is in fact a sandwich structure, as shown in Figure 9. The two outside layers are the polyimide films acting as the insulator, and a nickel-chromium alloy layer is conductive and is used to converts input voltages to thermal energy. The nickel-chromium alloy layer has very good thermal and electrical conductivity. The resistivity of the nickel-chromium alloy layer is $1\times 10^{-6}$(Ω·m), which has very good thermal and electrical conductivity. The thickness of the polyimide film used is 0.1 mm so that the film has good flexibility. In addition, the bending stiffness of polyimide film is much smaller than that of the pair of beams of the 3D U-shaped and the beam of Z-shaped actuator, so the polyimide film can be bend with deformation of the actuator beams and only bring little extra stiffness to the actuator. When a voltage is applied to the polyimide film which is intimately sticked to the beam surface, heat is generated within the polyimide film and then transferred to the beam to cause temperature rises. The external heat source is chosen due to the high electric conductivity of the beam (made of aluminum alloy AlSi10Mg), which will result in the short circuit if a voltage is directly applied to the actuator beam. Another important benefit of utilizing the external heat source is the conductive isolation of each pairs of beams since the metallic 3D microgripper is printed as a whole. This results in independently heating thus controlling each pair of beams. A controllable DC power supply is used to provide input voltages to the polyimide film. The average beam temperature is obtained by taking the average measured temperatures of five measuring points ($T_{1}$ through $T_{5}$ as shown in Figure 8a), which is evenly placed along the beam. In addition, the room temperature is 26.9°. The temperature is measured with the thermocouple. The displacement of the actuator tip is obtained via image processing under the microscopic view, as shown in Figure 8b. AlSi10Mg is used as the beam material considering its large thermal conductivity (203 W/(m·K)), large thermal expansion coefficient (23 × 10^−6^/°C), and small Young’s modulus (6 × 10^10^ Pa). 

In order to validate the theoretical model of the 3D U-shaped actuator established in Section 3, a group of different voltages (ranging from 9 V to 17 V, with an increment of 1 V) are applied to the polyimide films on one of the beam pairs of the 3D U-shaped actuator. The resulted displacements are measured through image processing, listed in Table 4. It is seen from Table 4 that the deviation between the analytical prediction and the measured results is around 11%. The deviations are calculated based on (30) as

(30) $$\delta_{2}\%=\frac{d_{Analytical}-d_{Measured}}{d_{Analytical}}\times 100\%$$

**Figure 10:**
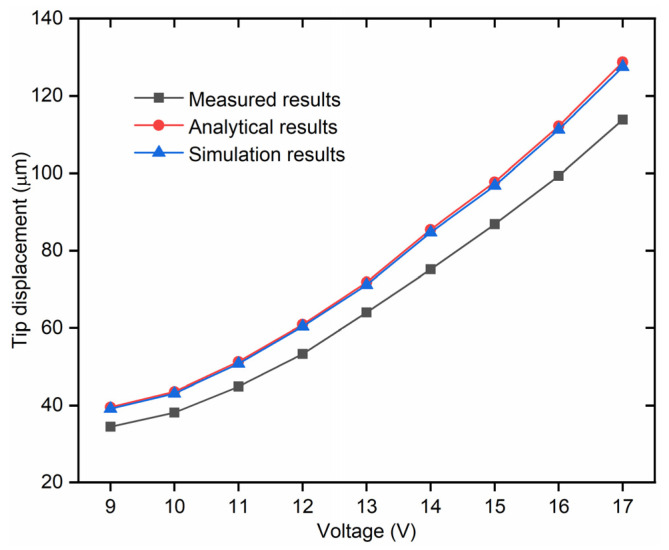
Measured, analytical and simulation output displacements of the actuator under the same actuation voltage.

The theoretical, testing and simulation results are also plotted as shown in Figure 10. As demonstrated in Section 4, the theoretical and simulation results agree well with each other, whereas the measured displacement is smaller with approximately 11% deviation. This can be explained by the fact that the temperature is measured only on the surface of the beam, and the temperature is used as the input to the system in the theoretical calculations and the simulations with FEM. However, the average temperature should be lower inside the beam, leading to the larger output displacements of the actuator in the results from theoretical analyses and FEM simulations. In addition, the temperature and displacement measurement method via the thermocouple and image processing respectively can induce system errors as well. The error can be reduced by measuring the displacement of the microgripper for many times and adopting a more accurate temperature measurement method.

## 6. Parameter Analyses of the 3D U-Shaped Actuator

Based on the theoretical model, it is possible to investigate toward how the actuator dimensions affect the output displacement thus optimizing the actuator design. Parameter analyses are conducted by calculating output displacements with varying one of the dimensions listed in Table 2 while keeping the rest dimensions unchanged. 

**Figure 11:**
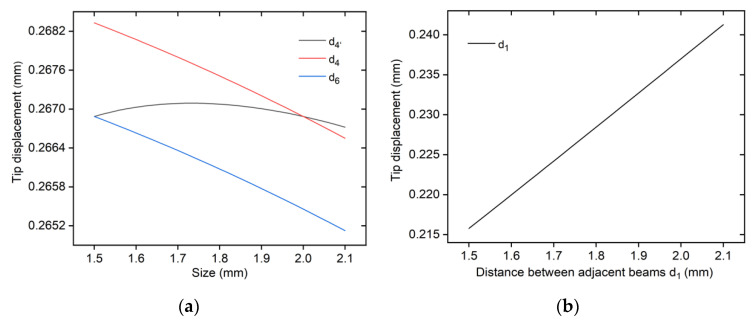

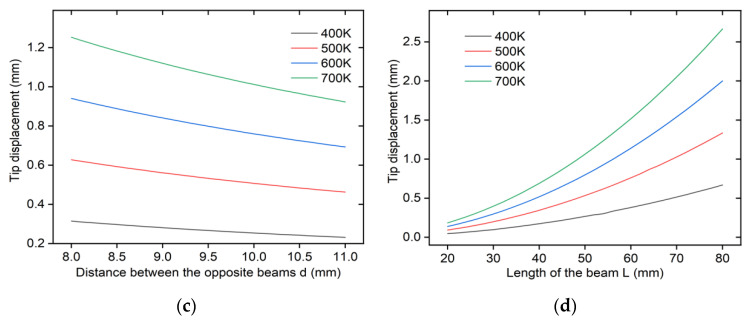
Parameter analyses of the 3D U-shaped actuator. (**a**) Output displacements vs. $d_{4}$, $d_{6}$, and $d_{4}$; (**b**) Output displacement vs. $d_{1}$; (**c**) Output displacement vs. d; (**d**) Output displacement vs. length of the beam L.

The schematic diagram of changing the width of the beams $d_{4}$, the thickness of the beams $d_{6}$, the distance between adjacent beams $d_{1}$, as well as the width $d_{4^{\prime }}$ of the beams but the area is unchanged is shown in Figure 11. In addition, $d_{4^{\prime }}$ is the width of the beam when the cross-sectional area of the beam remains unchanged in Figure 11.

It can be seen from Figure 11a,b that the output displacement decreases with increased of beam width $d_{4}$ and thickness $d_{6}$. If keeping the cross-sectional area unchanged, the displacement increases first followed by a decrease with increased of the beam width $d_{4}$. Besides, the output displacement increases with increasing the distance $d_{2}$ between adjacent beams.

The influence of the length L and the distance between the opposite beam pairs d on the output displacement is shown in Figure 11c,d. It can be seen from Figure 11c that the output displacement of the 3D U-shaped actuator increases with an increase of the beam length L. This is because, for the longer beam, the elongation of the beam at the same temperature will be larger, resulting in larger output displacement. 

Increasing the distance between the opposite beam pairs will result in smaller output displacement, as shown in Figure 11d. According to equation $I=b*h^{3}/12$, the increase of the section size of the beam will increase the inertia moment of the beam. In addition, as indicted in Equation (A3) in the Appendix A, the inertia moment is inversely proportional to the displacement caused by beam bending. The parameter analyses are summarized in Table 5.

## 7. Testing of the 3D Microgripper

### 7.1. Testing of the 3D U-Shaped Actuator

**Figure 12:**
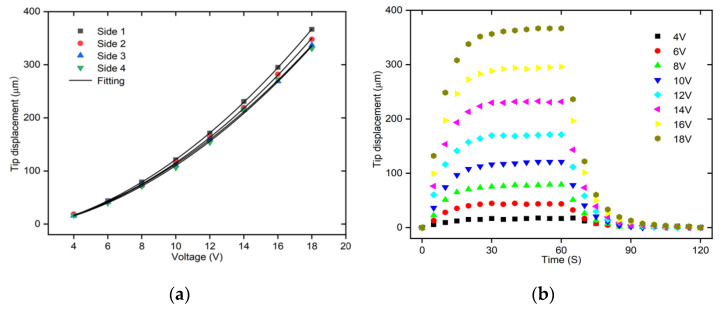
The static and dynamic testing of the 3D U-shaped actuator. (**a**)The static displacements vs. voltages of the 3D U-shaped actuator. (**b**) The dynamic testing of different sides of the side 1 of the 3D U-shaped actuator.

Detailed testing of each beam pair for the 3D U-shaped actuator is conducted, as shown in Figure 12a. It is found that the displacements for the four pairs of beams are fitted well with the second-order relations with the voltages. That is, the output displacement of the 3D U-shaped actuator is proportional to the square of the input voltages. The tip displacements of the beam pairs on side 1 and side 2 are larger than that of the beam pairs on side 3 and side 4, because the stiffness of the supporting base are different in the two actuating directions. The experimental results show that the output displacements of the 3D U-shaped actuator under 18 V are 366.68 μm, 348.07 μm, 336.91 μm, and 330.49 μm for the four beam pairs on side 1 to side 4, respectively. Dynamic responses of the first face of the 3D U-shaped actuator are also plotted as shown in Figure 12b. It is seen that the rise time for the 3D U-shaped actuator is approximately 30 s without an overshoot.

### 7.2. Testing of the Z-Shaped Actuator

**Figure 13:**
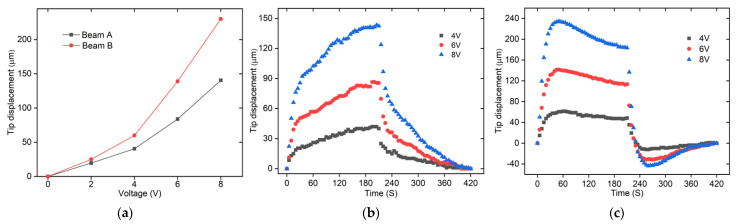
The static and dynamic testing of the Z-shaped actuator. (**a**) The static displacements vs. voltages of the Z-shaped actuator; (**b**) The dynamic displacements of the beam A; (**c**) The dynamic displacements of the beam B.

The static responses of the Z-shaped actuator are tested under different voltages (with an increment of 2 V), as shown in Figure 13a. Under 8 V, output displacements of the upper and lower beams (i.e., Beam A and Beam B, as shown in Figure 1a) of the Z-shaped actuator are 140.531 μm and 230.185 μm, respectively. The upper beam pair produces smaller displacement because it is nearer to the 3D U-shaped actuator, which makes heat dissipations more rapid. 

The dynamic response of the Z-shaped actuator is tested by applying a voltage to the polyimide film, holding for a period of time (210 s) and then removing the input voltages, as shown in Figure 13b,c. The experimental results show that the maximum displacements of beam A and beam B of the Z-shaped actuator under a voltage of 8 V are 143.32 μm and 233.59 μm respectively. In Figure 13c, when the power is turned off, the Z-shaped actuator first generates displacement in the recovery direction, and then generates displacement in the opposite direction. This phenomenon is caused by uneven heat dissipation of the microgripper. The rise time of the electrothermal actuators is a little bit indeed large and this can be further improved via feedback controls, which is part of our ongoing research.

## 8. Micro-Manipulation Experiments

**Figure 14:**
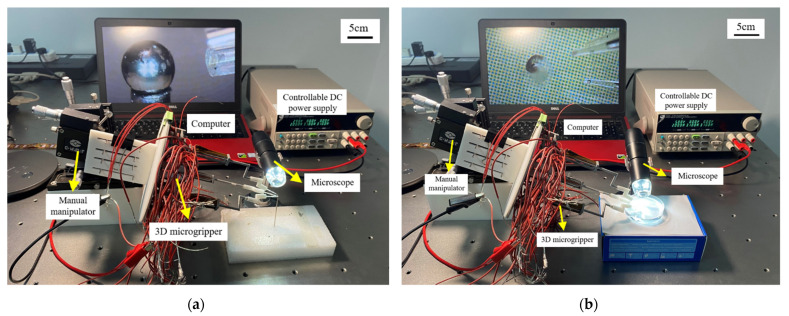
Experimental setup for manipulating (**a**) a micro ball and (**b**) the zebrafish embryo.

**Figure 15:**
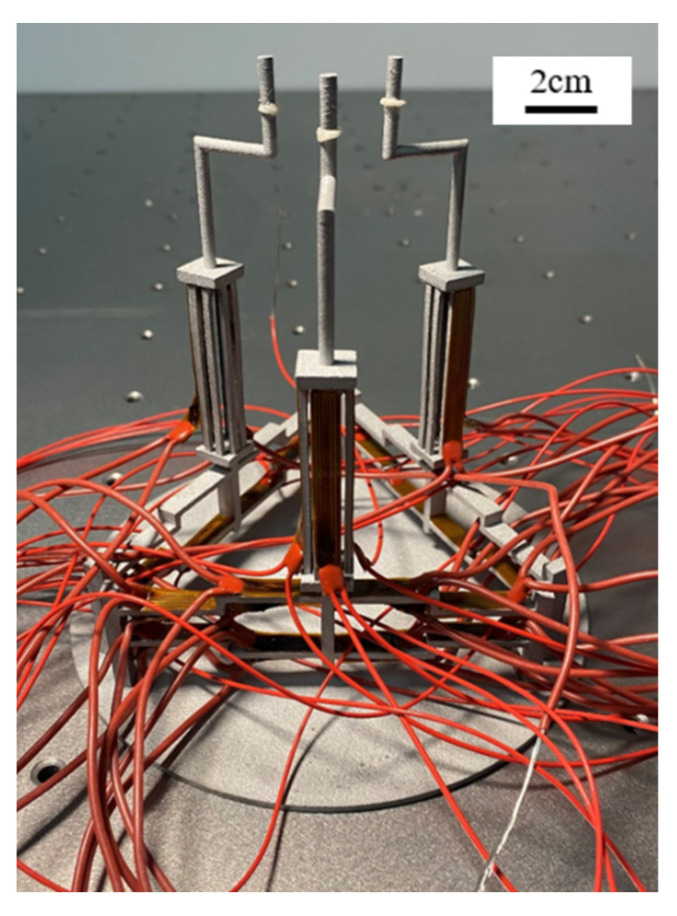
The fabricated 3D microgripper with the Polyimide films.

**Figure 16:**
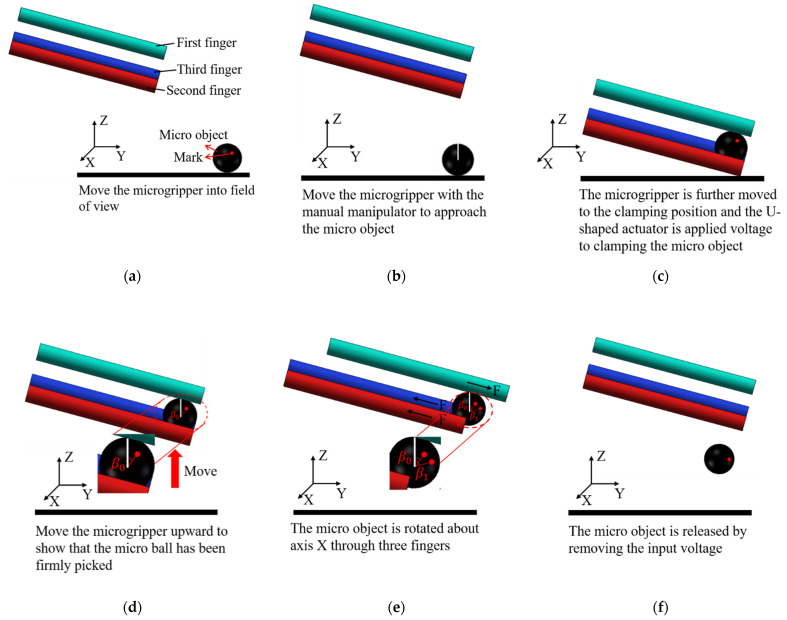
The schematic diagram of micro-objects manipulations. (**a**) Initial state; (**b**) Move the microgripper with the manual manipulator to approach the micro-object; (**c**) the micro-object is gripped; (**d**) Lift the micro-object; (**e**) Rotate the micro-object about an axis; (**f**) the micro-object is released.

**Figure 17:**
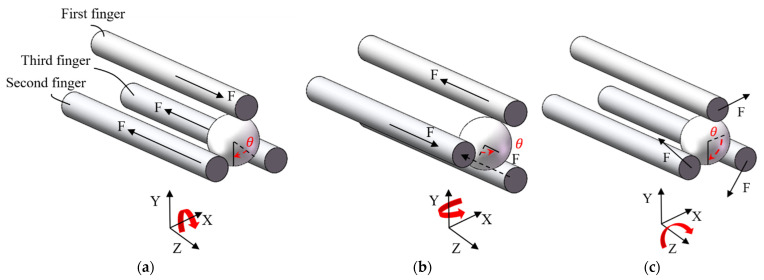
Rotations of micro object with the microgripper: (**a**) Rotation about axis X, (**b**) Rotation about axis Y, and (**c**) Rotation about axis Z.

The experimental setup for micromanipulation experiments is shown in Figure 14. The fabricated microgripper is mounted on a manual manipulator and the polyimide films are glued to beam surfaces for both the Z- and 3D U-shaped actuators. A controllable DC power supply is used to apply voltages to the polyimide films. In this experiment, a micro ball with a diameter of 2 mm and zebrafish embryos with a diameter of 600~800 μm are used. The fabricated 3D microgripper is shown in Figure 15. The bi-directional Z- and the 3D U-shaped actuator can join to provide three DOFs rendering the tip of each finger of the 3D microgripper is free to move in 3D space within the working space. Having three fingers, the 3D microgripper is not confined to only pick and place small/micro objects, but can freely rotate them in 3D space. The manipulation flowchart is illustrated in Figure 16. In this work, a metal micro ball with the diameter of 2 mm and zebrafish embryos with a diameter of around 600~800 μm are used for manipulations. The overall movement of the 3D microgripper is realized via a micromanipulator. Picking, releasing, and rotating manipulations are achieved with the 3D microgripper. The strategy of rotating a small/micro object is illustrated in Figure 17. With the proper collaboration of three fingers, both the small metal ball and the deformable zebrafish embryo can be rotated about XYZ axes, independently.

As shown in Figure 17a,b, to rotate a micro-object around axis X or Y, one of the fingers requires to move along the positive direction of axis Z, which is achieved by applying a voltage to the beam pair A of the corresponding Z-shaped actuator, and the other two fingers move in the negative direction of axis Z by heating the beam pair B. To rotate a micro-object about axis Z, the three fingers should move clockwise or anticlockwise by heating the 3D U-shaped actuators, as shown in Figure 17c. The rotation strategies will be validated in next section by conducting manipulation operations to a micro ball and a zebrafish embryo. Note that the manipulation is conducted in the open-loop manner by applying voltages to the corresponding beam pairs, i.e., with no feedback control involved. 

### 8.1. Manipulations of a Micro Ball

**Figure 18:**
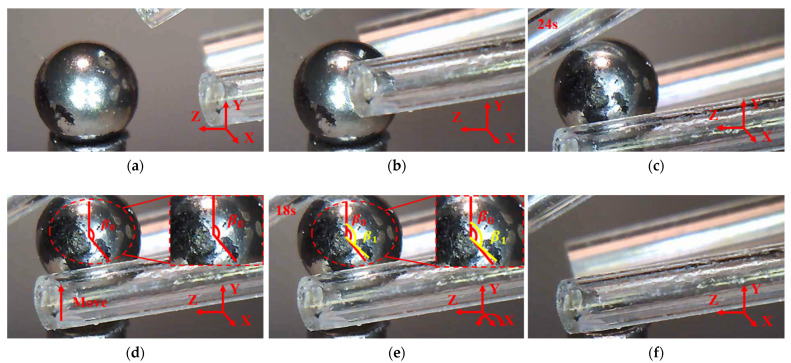
Manipulation of a micro ball: (**a**) Initial state; (**b**) the micro gripper tips are moved to enclose the micro ball; (**c**) the micro ball is gripped; (**d**) the micro ball is moved to a distance; (**e**) the micro ball is rotated about axis X; (**f**) the micro ball is released.

**Figure 19:**
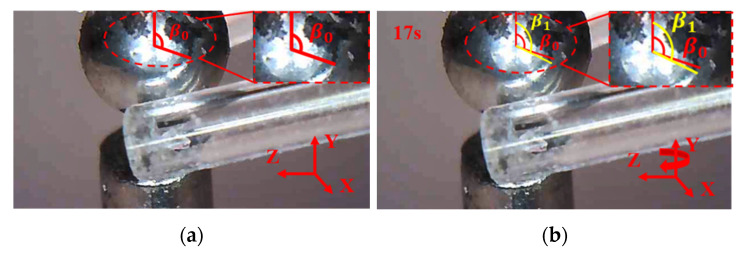
The micro ball is rotated about axis Y. (**a**) the micro ball is moved to a distance; (**b**) the micro ball is rotated about axis Y.

**Figure 20:**
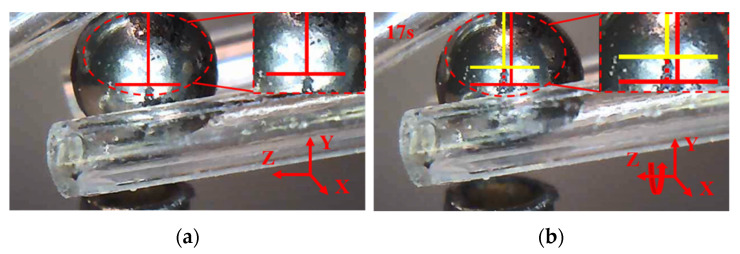
The micro ball is rotated about axis Z. (**a**) the micro ball is moved to a distance; (**b**) the micro ball is rotated about axis Z.

The manipulation of a micro ball begins by approaching and gripping the micro ball followed by rotating the micro ball about X, Y, and Z axes respectively, as shown in Figure 18, Figure 19 and Figure 20. In order to clearly observe the rotation of the micro ball, a mark was made on the surface of the micro ball. In Figure 18c, a red vertical line represents the reference line, and the other red line starts from the center of the micro ball and ends at the initial position of the marked point. The yellow line that connects the center of the micro ball and the mark point clearly indicates the rotation of the micro ball about axis X, as shown in Figure 18d. The mark position can then be represented by $\beta_{1}$ and the initial mark position by $\beta_{0}$. The procedure for picking, lifting and rotating the micro-ball is conducted with the following steps: (a) Move the microgripper into field of view; (b) Move the 3D microgripper with the manual manipulator to approach the micro ball; (c) Apply a voltage to the three 3D U-shaped actuators to clamp the micro ball; (d) Lift the micro-ball; (e) Rotate the micro ball about an axis; (f) Release the micro ball by removing all the input voltages. As show in Figure 19, the micro ball is rotated an angle about axis Y. Rotation about axis Z is indicated by the distance the mark point rises, as shown in Figure 20. The operation time is indicated in the Figures. Since the current manipulation is conducted in the manner of open loop, the time for operations can be large. Different from our previous work [32] where the V-shaped actuator is used instead of the Z-shaped actuator, the bi-directional Z-shaped actuator can provide both positive and negative actuation movement. In contrast, the V-shaped actuator can only move in one direction. The bi-directional actuation is extremely beneficial to dexterous micro manipulations and better mimics the human finger. 

### 8.2. Manipulations of a Zebrafish Embryo

**Figure 21:**
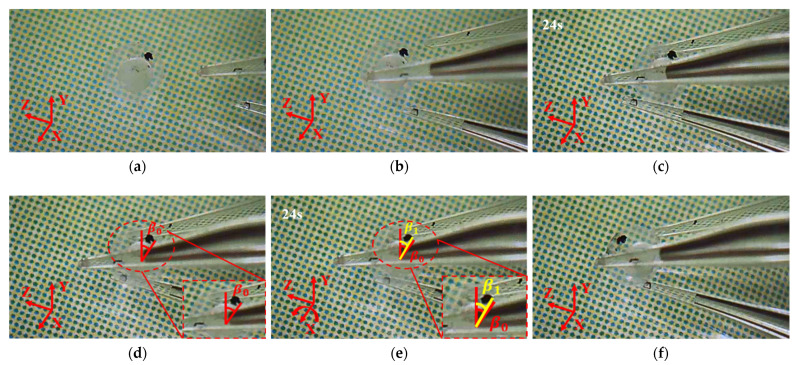
Manipulation of a zebrafish embryo: (**a**) Initial state; (**b**) approaching the micro embryo; (**c**) the embryo is gripped; (**d**) the embryo is moved to a distance; (**e**) the embryo is rotated about axis X; (**f**) the embryo is released.

**Figure 22:**
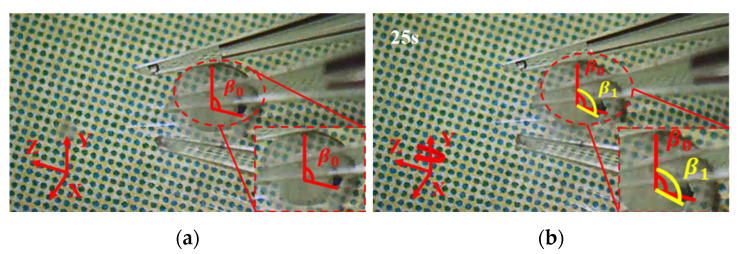
The zebrafish embryo is rotated about axis Y. (**a**) the zebrafish embryo is moved to a distance; (**b**) the zebrafish embryo is rotated about axis Y.

**Figure 23:**
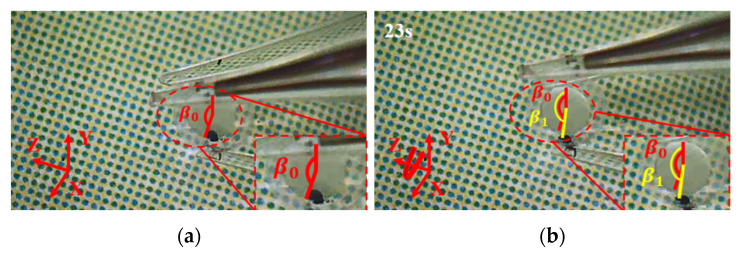
The zebrafish embryo is rotated about axis Z. (**a**) the zebrafish embryo is moved to a distance; (**b**) the zebrafish embryo is rotated about axis Z.

Following the same procedure and manipulation strategy, the manipulation of a zebrafish embryo is conducted as shown in Figure 21, Figure 22 and Figure 23. First, move the 3D microgripper to approach the zebrafish embryo with the micromanipulator; then, apply a voltage to the three 3D U-shaped actuators to clamp the zebrafish embryo. Now, the zebrafish embryo can be lifted and rotated. When finished, the embryo is released by removing the applied voltages. Note that releasing the embryo in liquid can be a little difficult as the embryo may adhere to the gripper tips. This can be prevented by releasing the embryo above the surface of the liquid so that the liquid tension is ruled out and gravity will drag the embryo to drop. 

## 9. Conclusions

In this work, we have developed a novel 3D three-fingered electrothermal microgripper. Each finger is composed of a bi-directional Z-shaped and a 3D U-shaped electrothermal actuator. The external heat source using the polyimide films is employed. The 3D U-shaped actuator can provide two DOFs and the Z-shaped actuator provides one DOF. Then each finger has three DOFs, which makes flexible 3D manipulation possible. The 3D microgripper is fabricated with metal 3D printing. The theoretical thermal-mechanical model of the 3D U-shaped actuator has been established, and has been verified with FEM simulations and further validated with experimental tests. A series of parameter analyses has also been conducted to provide insight and guidance in further improving the 3D microgripper design. Finally, micro-manipulations of a micro-ball and a zebrafish embryo, including picking, lifting, rotating, and releasing operations, have been carried out. Experimental results have demonstrated that the 3D microgripper proposed in this work is flexible and reliable for both basic picking and place and 3D rotations operations. In order to make the 3D microgripper more applicable, closed-loop control and force sensing techniques will be implemented, which is our ongoing work. 

## Figures and Tables

**Table 1:** Challenges and how to address them.

Challenge	Address the Challenge
A planar microgripper with multi-DOFs only brings the flexibility for the planar manipulation of microobjects	A three-finger microgripper that can operate micro objects in space is proposed.
A multi-finger microgripper has only one DOF for each finger	A three-finger microgripper with multiple DOFs for each finger is proposed.
Design and manufacture of the 3-fingers electrothermal microgripper	A novel 3D electro-thermal microgripper that can provide multi DOFs with three fingers is designed. Each finger is composed of a novel 3D U-shaped actuator and a bi-directional Z-shaped actuator. Besides, the microgripper is processed integrally by 3D printing.
The static theoretical model of 3D U-shaped actuator is deduced.	The theoretical relationship between the input temperature load and the output displacement of 3D U-shaped electrothermal actuator is deduced by using the superposition principle and deflection formula.
Measure the temperature of the beams of the 3D U-shaped actuator.	The average beams temperature is obtained by taking the average measured temperatures of many measuring points.
Due to manufacturing limitations, the dimension of the microgripper tip is larger than the micro object.	The glass capillary is assembled with the tip of the microgripper to operate the micro object.

**Table 2:** Dimensions of the 3D U-shaped electrothermal actuator (unit of length: mm).

Symbol	Definition	Value
d	Distance between two opposite beams	9.5
$d_{1}$	Distance between two adjacent beams in the different direction	2.75
$d_{2}$	Distance between two adjacent beams in the same direction	4
$d_{3}$	Distance between the beam and base boundary	3
$d_{4}$	Width of the beam	2
$d_{5}$	Length of base	12
$d_{6}$	Thickness of beam	1.5
$d_{7}$	Distance between beam and base boundary	0.5
$d_{8}$	Height of the base	3
L	Length of the beam	50

**Table 3:** Comparison between analytical and simulation results.

External Load (N)	Temperature (K)	Tip Displacement (mm)		Error (%)
		$d_{Analytical}$	$d_{ANSYS}$	
F = 0	400	0.2669	0.2649	0.7493
	500	0.5331	0.5298	0.6190
	600	0.7986	0.7947	0.4884
	700	1.0635	1.0596	0.3667
F = 0.1N	400	0.2649	0.2619	1.1325
	500	0.5311	0.5268	0.8096
	600	0.7966	0.7917	0.6151
	700	1.0615	1.0566	0.4616
F = K*Δ	400	0.2665	0.2647	0.6754
	500	0.5323	0.5293	0.5636
	600	0.7973	0.7940	0.4139
	700	1.0617	1.0586	0.2920

**Table 4:** Comparison of output displacement between analytical and experimental results.

Input Voltage (V)	Average Temperature Rise (°C)	Measured (μm)	Analytical (μm)	Deviation (%)
9	14.8	34.463	39.544	12.849
10	16.3	38.157	43.551	12.385
11	19.2	44.867	51.297	12.535
12	22.8	53.281	60.913	12.529
13	26.9	64.013	71.862	10.922
14	32	75.151	85.481	12.085
15	36.6	86.853	97.762	11.159
16	42	99.349	112.178	11.436
17	48.2	113.884	128.727	11.531

**Table 5:** Summary of the 3D U-shaped actuator parameter analysis.

Parameter	The Displacement
Length of beam L	Polynomially increase with L
Width of beam $d_{4}$	Polynomially decrease with $d_{4}$
Thickness of beam $d_{6}$	Polynomially decease with $d_{6}$
Distance from beam d	Polynomially decease with d
Distance between adjacent beams $d_{1}$	Linearly increase with $d_{1}$
When the area is constant, the width of the beam $d_{4^{\prime }}$	Polynomially increasing first and then decreasing with $d_{4^{\prime }}$

**Figure A1:**
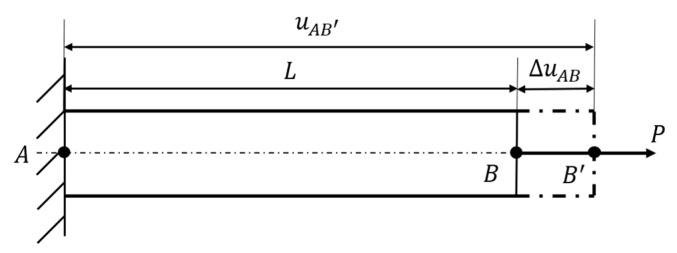
Elongation of a beam.

**Figure A2:**
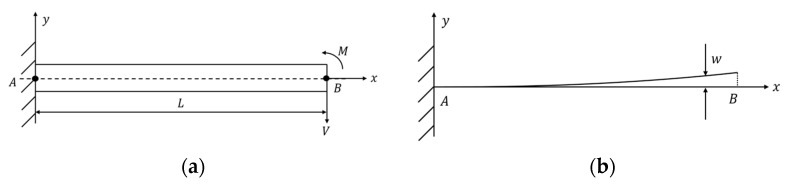
Deflection of a cantilever beam: (**a**) the cantilever beam subjected to bending moment and shear force; (**b**) deflection curve of the cantilever beam.

## Data Availability

The data presented in this study are available in supplementary material here.

## Supplementary Materials

The following are available online at https://www.mdpi.com/article/10.3390/mi12121512/s1.

## Appendix A. Elongation and Deflection of a Cantilever Beam

Elongation of a cantilever beam subjected to a force acting at the free end, as shown in Figure A1, is calculated as in (A1).

(A1) $$\Delta u_{AB}=\frac{PL}{EA}$$

where *E* and *A* are the Young’s modulus and the cross-sectional area of the beam. *L* is the original length of the beam. Then, the elongated beam length is calculated as in (A2).

(A2) $$u_{AB^{\prime }}=L+\Delta u_{AB}$$

Consider a cantilever beam with the concentrated loads, the bending moment M and the shear force V, acting at the free end, as shown in Figure A2a. The deflection curve of the cantilever beam is shown in Figure A2b, in which w(x) refers to the deflection of the beam, i.e., the displacement in the y direction of any point on the axis of the beam.

The basic differential equation of the deflection curve of a beam is

(A3) $$\frac{d^{2}w}{dx^{2}}=\frac{M\left(x\right)}{EI}$$

in which *M*(*x*) is the bending moment, *E* is the Young’s modulus of the beam material and *I* is the moment of inertia of the cross-sectional area of the beam with respect to the neutral axis. The bending moment at any cross-sectional area is calculated as

(A4) M(x)=M−V·(L−x)

Combining (A1) and (A2), we have

(A5) $$\frac{d^{2}w}{dx^{2}}=\frac{M-V\cdot \left(L-x\right)}{EI}$$

Integrating (A3) on the beam axis gives

(A6) $$w^{\prime }\left(x\right)=\frac{M-VL}{EI}x+\frac{V}{2EI}x^{2}$$

Then, for the cantilever beam shown in Figure A2 deflection of the beam and slope of the deflection curve at the free end can be obtained as in (A7).

(A7) $$w^{\prime }\left(L\right)=\frac{ML}{EI}-\frac{VL^{2}}{2EI}$$

Further integrate (A6) along the beam axis gives

(A8) $$w\left(x\right)=\frac{M-VL}{2EI}x^{2}+\frac{V}{6EI}x^{3}$$

For the cantilever beam shown in Figure A2 deflection of the beam at the free end is derived as in (A9).

(A9) $$w\left(L\right)=\frac{ML^{2}}{2EI}-\frac{VL^{3}}{3EI}$$
